# Supplementary material for: MetaRibo-Seq measures translation in microbiomes
Source: Nat Commun. 2020 Jun 29;11:3268. doi: 10.1038/s41467-020-17081-z (PMC7324362; doi:10.1038/s41467-020-17081-z)
Supplement: Supplementary file 10 — Supplementary Data 7 [file 41467_2020_17081_MOESM10_ESM.zip › File2/Confidence_High_Taxonomy/244666_out.krona.html]

Javascript must be enabled to view this page.

members
magnitude
magnitudeUnassigned
count
unassigned
taxon
rank

244666\_out

2

superkingdom
2
2

203691
1
phylum

1
203692
class

order
136
1

1951287
1
species

SRS049959\_contig\_number\_contig-100\_1163.294620

phylum
976
1

1
200643
class

171549
1
order

family
171552
1

1
838
genus

1
2292365

SRS015794\_contig\_number\_contig-100\_226.90060
species
